# Recruitment of M1 Macrophages May Not Be Critical for Protection against Colitis-Associated Tumorigenesis

**DOI:** 10.3390/ijms222011204

**Published:** 2021-10-18

**Authors:** Itzel Medina-Andrade, Jonadab E. Olguín, Stephanie Guerrero-García, Jossael A. Espinosa, Elizabeth Garduño-Javier, Victoria Hernández-Gómez, Felipe Vaca-Paniagua, Miriam Rodríguez-Sosa, Luis I. Terrazas

**Affiliations:** 1 Laboratorio Nacional en Salud FES-Iztacala, Universidad Nacional Autónoma de México, Av. De los Barrios 1, Los Reyes Iztacala, Tlalnepantla 54090, Estado de México, Mexico; itzelmedina.andrade@iztacala.unam.mx (I.M.-A.); je.olguin@iztacala.unam.mx (J.E.O.); stephanieg1721@gmail.com (S.G.-G.); jossa.espinosa@comunidad.unam.mx (J.A.E.); elizabethgj@comunidad.unam.mx (E.G.-J.); victoriahg.29@comunidad.unam.mx (V.H.-G.); felipe.vaca@gmail.com (F.V.-P.); rodriguezm@unam.mx (M.R.-S.); 2 Unidad de Biomedicina, Facultad de Estudios Superiores (FES)-Iztacala, Universidad Nacional Autónoma de México, Av. De los Barrios 1, Los Reyes Iztacala, Tlalnepantla 54090, Estado de México, Mexico

**Keywords:** colitis-associated colon cancer, inflammation, IL4Rα, macrophages

## Abstract

A close connection between inflammation and the risk of developing colon cancer has been suggested in the last few years. It has been estimated that patients diagnosed with some types of inflammatory bowel disease, such as ulcerative colitis or Crohn’s disease, have up to a 30% increased risk of developing colon cancer. However, there is also evidence showing that the activation of anti-inflammatory pathways, such as the IL-4 receptor-mediated pathway, may favor the development of colon tumors. Using an experimental model of colitis-associated colon cancer (CAC), we found that the decrease in tumor development in global IL4Rα knockout mice (IL4RαKO) was apparently associated with an inflammatory response mediated by the infiltration of M1 macrophages (F480^+^TLR2^+^STAT1^+^) and iNOS expression in colon tissue. However, when we developed mice with a specific deletion of IL4Rα in macrophages (LysMcreIL4Rα^−/lox^ mice) and subjected them to CAC, it was found that despite presenting a large infiltration of M1 macrophages into the colon, these mice were as susceptible to colon-tumorigenesis as WT mice. These data suggest that in the tumor microenvironment the absence of IL4Rα expression on macrophages, as well as the recruitment of M1 macrophages, may not be directly associated with resistance to developing colon tumors. Therefore, it is possible that IL4Rα expression in other cell types, such as colonic epithelial cells, could have an important role in promoting the development of colitis-associated colon tumorigenesis.

## 1. Introduction

Around 14.1 million new cases of cancer are diagnosed worldwide every year. Among the five types of cancer with the highest incidence and mortality is colorectal cancer (CRC) [1]. The development of CRC has different origins, though between 15% and 20% of CRC cases have been associated with inflammatory processes, mainly with inflammatory bowel diseases (IBD), such as ulcerative colitis (UC) and Crohn’s disease (CD) [2]. In fact, it has been estimated that patients diagnosed with some type of IBD have a higher risk of developing colitis-associated colon cancer (CAC), as there are findings suggesting that inflammation can help incipient neoplasms to acquire different abilities to grow [3]. By contrast, several studies have shown that an anti-inflammatory response, mediated by interleukin-4 receptor expression (IL4R), may promote tumor cell survival and proliferation [4,5]. The IL-4 receptor controls the signaling of anti-inflammatory/Th2 responses and the IL-4 signaling pathway functions through two types of receptors [6]. The type I receptor signals exclusively for IL-4 and is mainly expressed in bone marrow-derived cells. It is composed of an IL-4 receptor α-chain (IL4Rα) and a γ-chain common. Signaling through the type II receptor is mediated by both IL-4 and IL-13, and is mostly expressed in cells that are not derived from bone marrow. It is composed of an IL-13 receptor α1 chain (IL13Rα1) and IL4Rα [7]. Both type I and type II receptors have in common the downstream signaling pathway of the transcription factor STAT6, which forms homodimers that translocate to the nucleus and promote the biological functions of IL-4 [7]. In the immune system, IL4Rα expression in macrophages and their interaction with anti-inflammatory signals mediated by IL-4, IL-13 and TGF-β can promote their activation toward alternatively activated macrophages (M2). These M2 macrophages participate in wound repair and share characteristics with tumor-associated macrophages (TAMs). On the other hand, macrophages can also be activated towards an inflammatory profile or classical activation (M1), depending on interaction with bacterial components, such as LPS, or inflammatory signals mediated by IFN-γ. 

In colon cancer, macrophages are one of the main immune cells infiltrated in this type of tumor [8]. However, the infiltration of these macrophages has been associated with poor survival in patients [8], since such macrophages can acquire a TAM phenotype promoting tumorigenic functions, such as immunosuppression, angiogenesis, and extracellular matrix remodeling, which favors tumor growth and establishment [9,10,11]. It has also been shown that M1 macrophages may have tumoricidal ability [12] through inducible nitric oxide synthase (iNOS) [13,14,15] expression, activation of cytotoxic T CD8^+^ cells [16,17] and production of TNF-α [18]. In fact, there are new proposed therapies to treat different tumors focused on the suppression of either TAM or M2 polarization and therefore enhancement of M1 activity [19]. Nevertheless, the density of M1 macrophages infiltrated into the tumor in colon cancer is much lower than that of TAMs [20], whose profile is favored by IL4Rα expression, as well as by IL-4 from tumor cells in the tumor microenvironment [5,21]. In the present work, we evaluated whether, in a murine model of CAC, the absence of IL4Rα, and therefore TAMs, could be associated with lower colon tumorigenesis due to a tumoricidal response mediated by M1 macrophages, whose activation profile would be favored by the absence of IL4Rα.

## 2. Results

### 2.1. The Complete Absence of the IL-4 Receptor α-Chain Inhibits Colon Tumor Development

**Figure 1:**
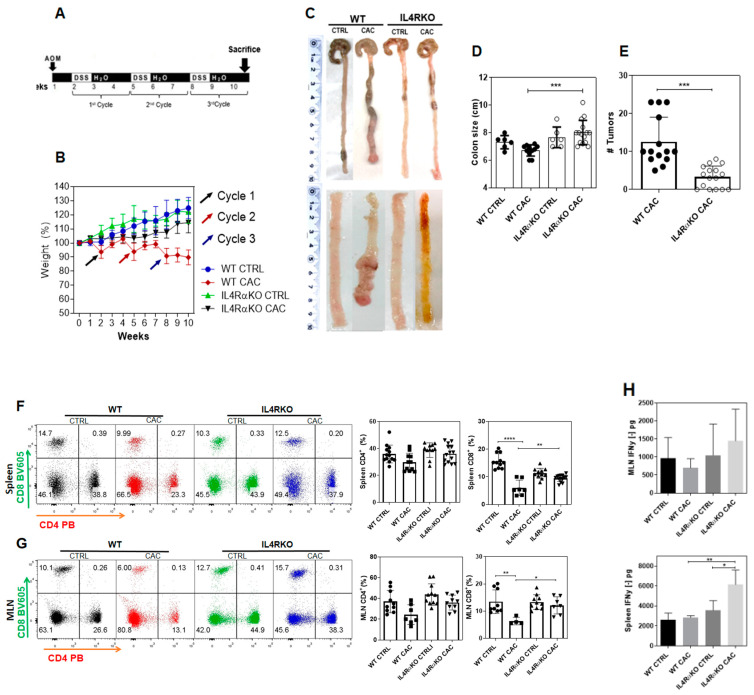
The absence of the IL4 receptor alpha inhibits colon tumorigenesis. (**A**) The AOM/DSS model. (**B**) Loss of weight per week expressed as percentage since the induction with AOM until the end of the third cycle with DSS in WT and IL4RαKO, CTRL and CAC mice. (**C**) Representative photographs of colon length of WT- and IL4RαKO-CAC mice at the end of the third DSS cycle, showing the colon from the cecum to the rectal region and the colon without cecum and with longitudinal section. (**D**) Colon size. (**E**) Number of tumors present in the distal region of the colon. Dot plots and graphs of total percentages of T CD4^+^ and CD8^+^ cells in (**F**) spleen and (**G**) mesenteric lymph nodes (MLN) of WT and IL4RαKO mice. (**H**) 1 × 10^5^ spleen and MLN cells were stimulated with plate-bound anti-CD3 antibody for 48 h to analyze in supernatants IFN-γ production using a CBA Mouse Inflammation Kit. The data came from three different experiments with at least three mice per group. A one-way ANOVA was performed for all the panels and the mean was presented with standard deviation for each experimental group. Significance values: *p* ≤ 0.05 (*), *p* ≤ 0.01 (**), *p* ≤ 0.001 (***), *p* ≤ 0.0001 (****).

Previous studies have suggested that IL4Rα is involved in promoting tumor development in colon cancer [4,5]. To confirm this, we used an AOM/DSS mouse model (Figure 1A) for the induction of CAC in IL4Rα global knockout mice (IL4RαKO-CAC). Initially, we monitored weekly the weight of mice after the administration of AOM until the end of the third DSS cycle. Notably, IL4RαKO-CAC mice did not show any weight loss during the three cycles of DSS as compared to WT-CAC mice that lost up to 20% of their weight (Figure 1B). Consistent with this observation, the expected shortened colon was not observed in IL4RαKO-CAC mice (Figure 1C,D), and this group also displayed significantly fewer tumors than WT-CAC mice (Figure 1C,E); these tumors were smaller and there were even tumor-free IL4RαKO-CAC mice (Figure 1E). 

These results indicate that IL4Rα expression was directly related to tumor development in CAC. In order to define the impact of the absence of IL4Rα in our CAC model, we decided to explore the populations of CD4 and CD8 T cells at spleen and mesenteric lymph nodes (MLN). We did not find differences in percentages of CD4^+^ T cells between IL4RαKO-CAC mice and WT-CAC mice (Figure 1F,G). In contrast, a remarkable change was observed in the percentage of CD8^+^ T cells. Whereas CAC induced a significant reduction on CD8^+^ T cells at both the spleen and MLN in WT mice, we did not find a decrease in this cell population in IL4RαKO-CAC mice (Figure 1F,G). IFN-γ is a cytokine involved in the efficient function of CD8^+^ T cells and in M1 macrophage polarization [22]. Therefore, we evaluated whether in IL4RαKO-CAC mice lymphocytes may induce the activation of tumoricidal phenotypes in M1 macrophages through the production of IFN-γ. IFN-γ production by total cells from MLNs and spleen cells stimulated with anti-CD3 antibody was determined. We did not find differences in IFN-γ production in MLN cells (Figure 1H upper panel). However, spleen cells from IL4RαKO-CAC mice displayed higher production of IFN-γ compared to WT-CAC mice (Figure 1H, bottom panel). These data suggest that IL4RαKO mice have a higher inflammatory profile, which is probably associated with the lower number of colon tumors observed.

### 2.2. The Absence of IL4Rα Favors M1 Macrophage Recruitment in the Colon 

**Figure 2:**
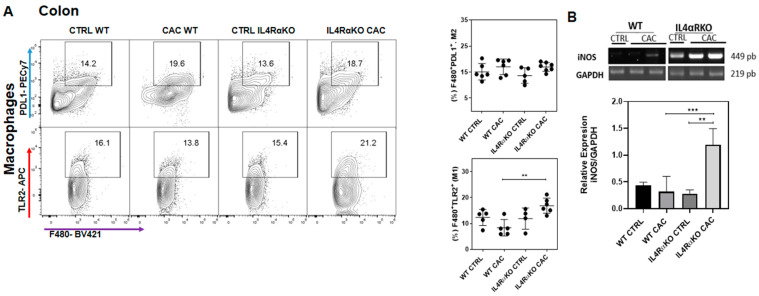

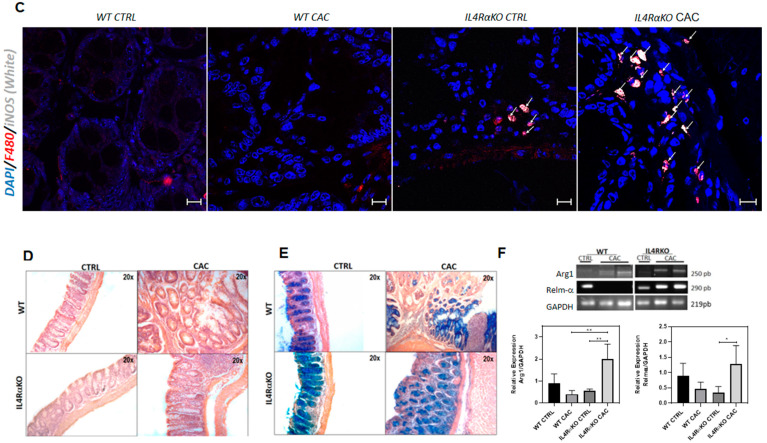
The absence of IL4Rα promotes M1 macrophages recruitment and a protective inflammatory response. (**A**) Flow cytometry, dot plots and graphs representing the percentage of M1 macrophages (F480^+^TLR2^+^) and M2 macrophages (F480^+^PDL1^+^) infiltrated into the colon in WT and IL4RαKO mice, euthanized at the end of the third DSS cycle. (**B**) Representative and total iNOS RT–PCR data from colon samples normalized with GAPDH constitutive gene. (**C**) Confocal representative merged image of immunofluorescence staining of colon tissue of WT-CTRL, WT-CAC, IL4RαKO-CTRL and IL4RαKO-CAC mice using DNA-binding dye (DAPI) in blue, F4/80 in red and iNOS in white. Photographs were taken with a 63X objective. (**D**,**E**) Hematoxylin and eosin (H&E) and Alcian blue staining of distal colon region slices in WT and IL4RαKO mice. (**F**) Representative and total RT–PCR data for Arg1 and Relm-α expression from colon samples normalized with the GAPDH constitutive gene. The data came from two different experiments with at least three mice per group. A one-way ANOVA was performed for all the panels and the mean was presented with standard deviation for each experimental group. Significance value *p* ≤ 0.05 (*), *p* ≤ 0.01 (**), *p* ≤ 0.001 (***). Scale bars indicates 10 μm.

To determine whether M1 or M2 macrophages play a role in tumor reduction in IL4RαKO-CAC mice, we evaluated colon M1-markers of classically activated macrophages through Toll like receptor 2 (TLR2) expression [23], as well as M2 markers through Programed Death Ligand 1 (PDL1) expression [24]. We found that the percentage of M1 macrophages F4/80^+^TLR2^+^ infiltrated in the colon were higher in IL4RαKO-CAC mice compared to WT-CAC mice (Figure 2A). However, we did not find differences in the percentage of F4/80^+^PDL1^+^ M2 macrophages infiltrated into the colon (Figure 2A). These data suggested that M1 macrophages might be involved in tumor reduction in IL4RαKO-CAC mice. Therefore, we decided to evaluate whether M1 macrophages could be expressing genes associated with tumor reduction, such as nitric oxide synthase (iNOS), since several reports have shown that iNOS overexpression can exert cytotoxic effects on tumor cells [14,25,26]. We found that IL4RαKO-CAC mice displayed a significant overexpression of the iNOS gene in colon tissue compared to WT-CAC mice (Figure 2B). Furthermore, by immunofluorescence of colon tissue we confirmed that macrophages that infiltrated the colon of IL4RαKO-CAC mice were M1 macrophages (F480^+^iNOS^+^) (Figure 2C; Supplementary Figure S1). Next, we evaluated whether the inflammatory response observed in IL4RαKO-CAC mice may generate tissue damage. The structure and architecture of the intestinal epithelium (Figure 2D), as well as mucus production by goblet cells, were well preserved in IL4RαKO-CAC mice, while WT-CAC mice had severe damage in colonic tissue with altered colon structure and loss of goblet cells (Figure 2E). In addition, colon tissue from IL4RαKO-CAC mice overexpressed Arg1 and Relmα1 (Figure 2F), which are genes associated with tissue-damage repair caused by early inflammatory processes [27]. 

### 2.3. IL4Rα Expression in Macrophages Is Not Directly Related to Tumor Development in CAC

**Figure 3:**
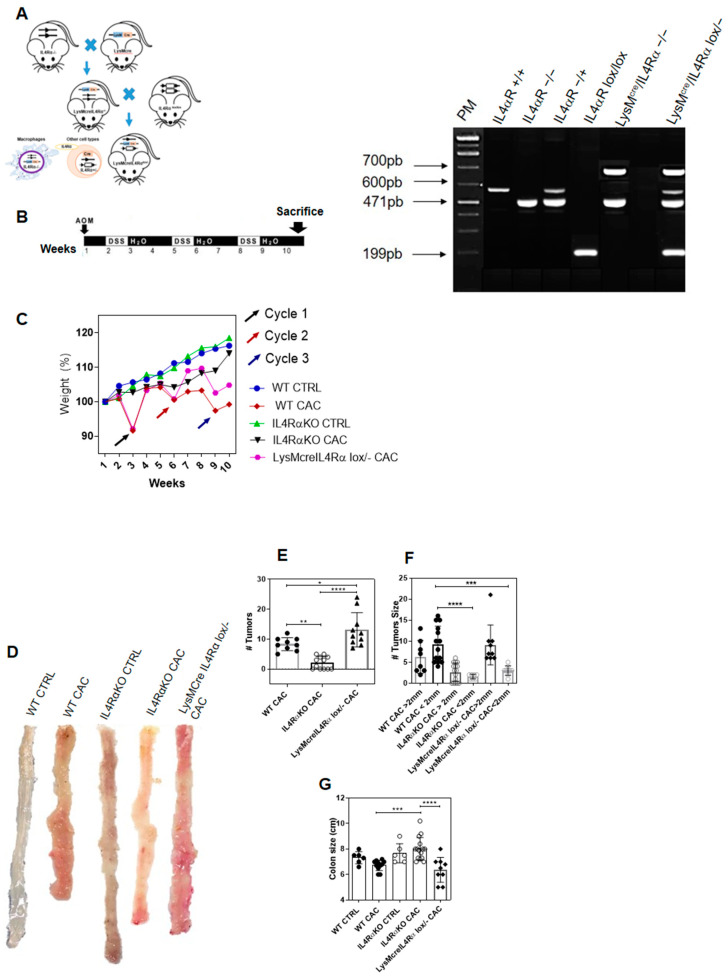
Lack of expression of IL4Rα in macrophages is not directly related to protection in CAC. (**A**) IL4Rα and BALB/c mice were crossed with mice expressing LysMcre and IL4Rα lox/lox to produce LysMcreIL4Rα^−/lox^ BALB/c mice. (**B**) Genotyping of LysMcreIL4Rα^−/lox^ mice. The PCR of IL4Rα is 471 base pairs, loxP is 190 base pairs (floxed) and specific Cre is 700 base pairs. (**C**) Weight per week expressed in percentage form since AOM induction until the end of the third DSS cycle in WT, IL4RαKO and LysMcreIL4Rα^−/lox^ mice. (**D**) Representative photographs of whole colon from cecum to rectum and longitudinal section of colon without cecum and free of fecal matter in WT, IL4RαKO and LysMcreIL4Rα^−/lox^ mice with CAC. (**E**) Colon size. (**F**) Number of tumors in colon. (**G**) Tumor size, <2 mm and >2 mm. The data came from three different experiments with at least three mice per group. A one-way ANOVA was performed for all the panels and the mean was presented with standard deviation for each experimental group. Significance value *p* ≤ 0.05 (*), *p* ≤ 0.01 (**), *p* ≤ 0.001 (***), *p* ≤ 0.0001 (****).

We found that in the absence of IL4Rα, the recruitment of M1 macrophages in the colon apparently had an important role providing protection during CAC. However, we did not know if the protective role of M1 macrophages and the inhibition of colon tumorigenesis in IL4RαKO-CAC mice could be related to the absence of IL4Rα in macrophages and therefore the absence of a TAM phenotype. To test this, we developed a cell-line specific knockout mouse in the IL4Rα chain in macrophages (LysMcreIL4Rα^−/lox^), as previously reported [28] (Figure 3A; Supplementary Figure S2). These specimens were then subjected to the AOM/DSS model of colon cancer (Figure 3B). We observed that both WT mice and LysMcreIL4Rα^−/lox^ mice lost weight at the end of each DSS cycle (Figure 3C). In addition, the colons of LysMcreIL4Rα^−/lox^-CAC mice were shortened, as the colons of WT-CAC mice, whereas IL4RαKO-CAC mice maintained a normal weight and colon size as healthy mice (Figure 3C–E). Furthermore, LysMcreIL4Rα^−/lox^-CAC mice developed tumors in similar numbers to WT-CAC mice; however, colon tumors were smaller in LysMcreIL4Rα^−/lox^-CAC mice (Figure 3D,G). These results suggest that macrophages lacking IL4Rα may not be directly responsible for the inhibition of tumor development in IL4RαKO-CAC mice.

### 2.4. Colon Infiltration of M1 Macrophages in LysMcreIL4Rα^−/lox^-CAC Mice Is Not Enough to Avoid Colon Tumorigenesis

**Figure 4:**
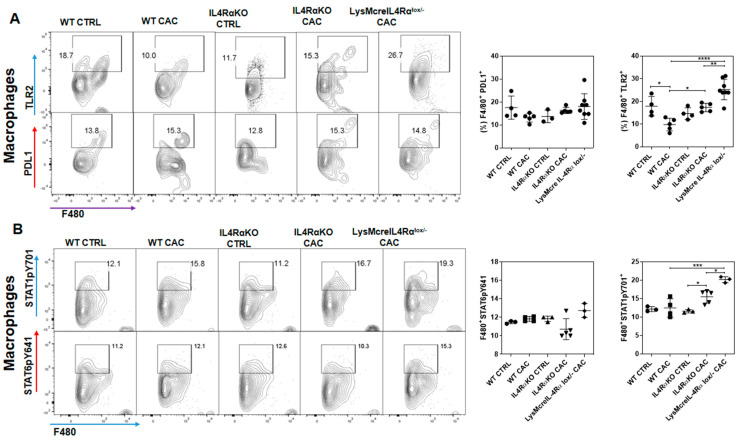

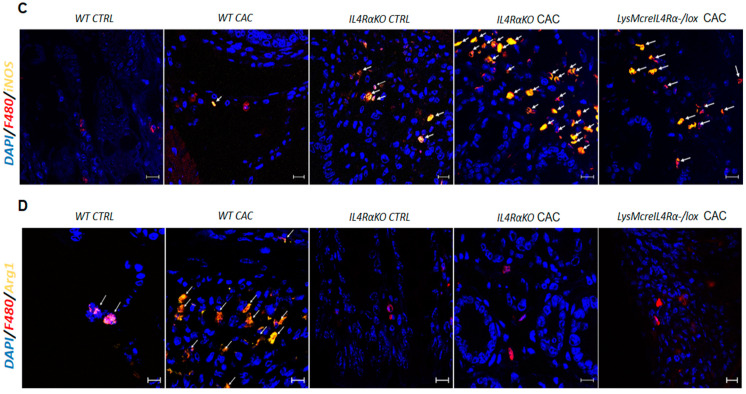
Infiltration of M1 macrophages in LysMcreIL4Rα^−/lox^ -CAC mice is not enough to avoid colon tumorigenesis. (**A**) Flow cytometry of colon cells, dot plots and graphs representing the percentage of macrophages M1 (F480^+^TLR2^+^), M1 (F480^+^pSTAT1^+^) and (**B**) macrophages M2 (F480^+^PDL1^+^), M2 (F480^+^pSTAT6^+^) infiltrated into the colon of WT-, IL4RαKO- and LysMcreIL4Rα^−/lox^-CAC mice, euthanized at the end of third DSS cycle. (**C**) M1 macrophage activation markers in colon tissue assessed by immunofluorescence. Microscopy data of colon sections from WT-CTRL, WT-CAC, IL4RαKO CTRL, IL4RαKO-CAC and LysMcreIL4Rα^−/lox^-CAC mice, stained with the DNA-binding dye (DAPI) in blue, F480 in red and iNOS in yellow. (**D**) M2 macrophage activation markers in colon tissue assessed by immunofluorescence. Microscopy data of colon sections from WT-CTRL, WT-CAC, IL4RαKO-CAC and LysMcreIL4Rα^−/lox^ -CAC, stained with the DNA-binding dye (DAPI) in blue, F480 in red and Arg1 in yellow. Photographs were taken with a 63X objective. The data came from three different experiments with at least three mice per group. A one-way ANOVA was performed for all the panels and the mean was presented with standard deviation for each experimental group. Significance value *p* ≤ 0.05 (*), *p* ≤ 0.01 (**), *p* ≤ 0.001 (***), *p* ≤ 0.0001 (****). Scale bars indicates 10 μm.

Next, we evaluated the activation profile of macrophages infiltrated into the colon. We found that in LysMcreIL4Rα^−/lox^-CAC mice there was a higher percentage of M1 TLR2^+^ macrophages (Figure 4A), but we did not observe differences in the percentage of M2 macrophages PDL1^+^ compared to WT-CAC mice (Figure 4A). To evaluate the functionality in the signaling pathway of either M1 or M2 macrophages in the colon, we analyzed the phosphorylation of the transcription factor STAT1 (pSTAT1), which is associated with an inflammatory response and with M1 activation, as well as the phosphorylation of the transcription factor STAT6 (pSTAT6) downstream of IL4Rα, which is associated with an anti-inflammatory response and with the activation of M2 macrophages. The percentage of M1 STAT1^+^ macrophages infiltrated into the colon was high in IL4RαKO-CAC mice; however, LysMcreIL4Rα^−/lox^-CAC mice displayed the highest recruitment of M1 STAT1^+^ macrophages into the colon, while the percentage of M2 STAT6^+^ macrophages did not change compared to IL4RαKO-CAC and WT-CAC mice (Figure 4B). In addition, by confocal immunofluorescence, we confirmed that both LysMcreIL4Rα^−/lox^-CAC mice and IL4RαKO-CAC mice displayed M1 macrophages (F480^+^iNOS^+^) infiltrated into the colon (Figure 4C; Supplementary Figure S3); however, M2 macrophages (F480^+^Arg1^+^) were not observed in the same groups, whereas WT-CAC mice recruited higher numbers of M2 macrophages (Figure 4D; Supplementary Figure S4). These data indicate that the high infiltration of M1 macrophages in LysMcreIL4Rα^−/lox^-CAC mice seems to be not enough to prevent colon tumorigenesis. 

### 2.5. M1 Macrophages in LysMcreIL4Rα^−/lox^ Mice Do Not Have a Protective Role during CAC

**Figure 5:**
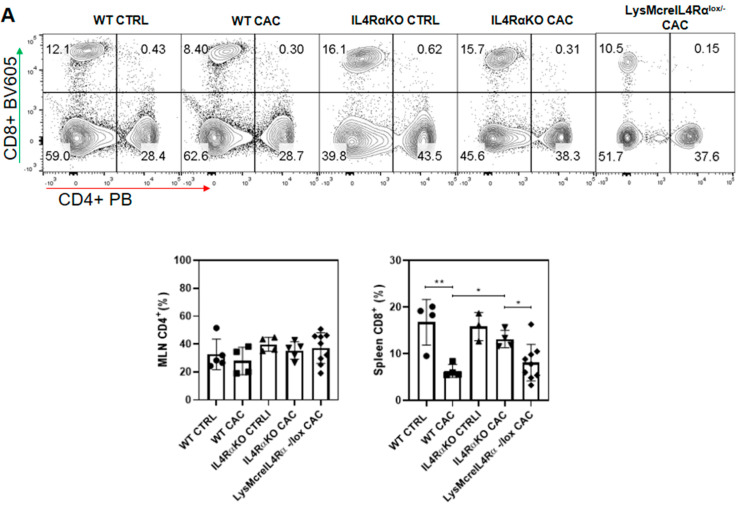

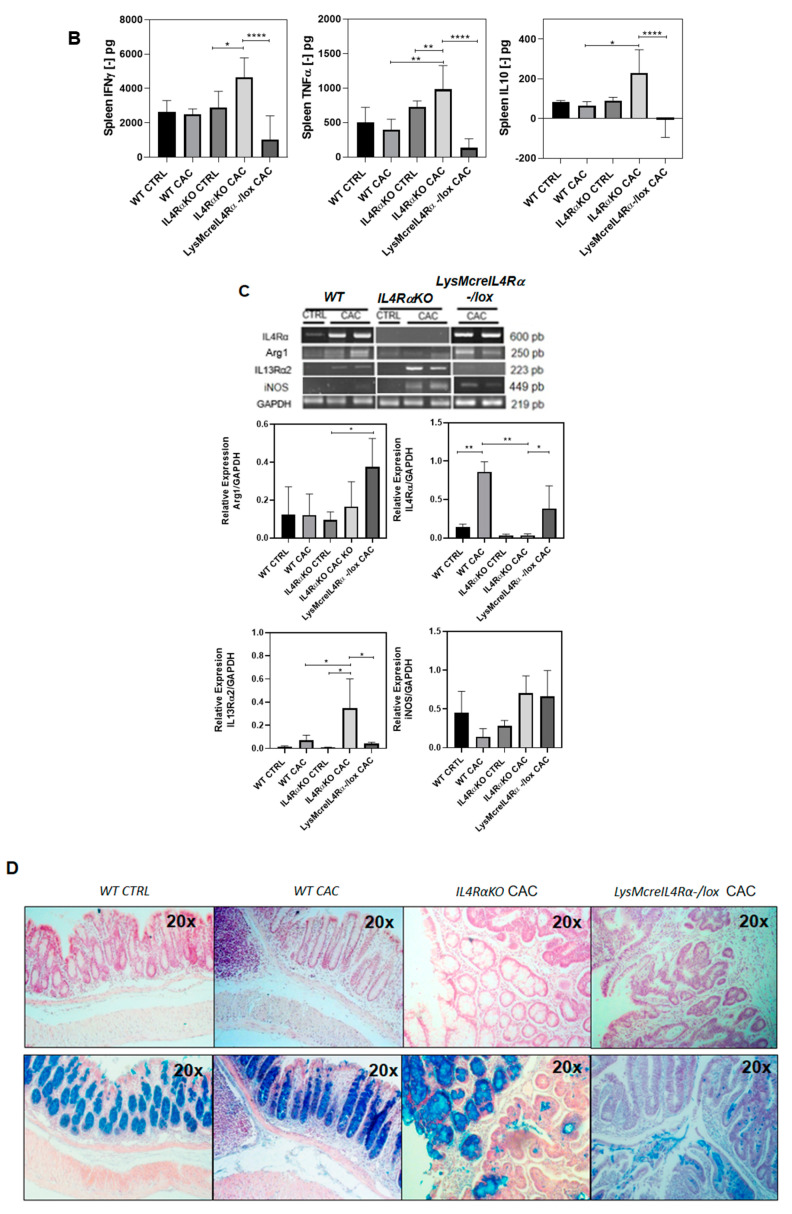

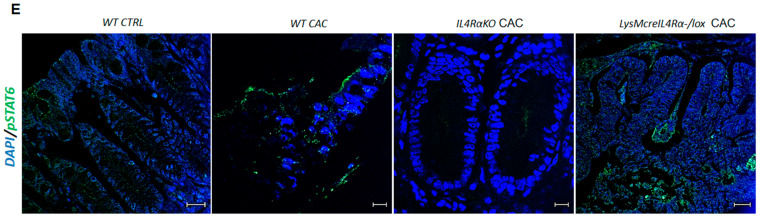
M1 macrophages in LysMcreIL4Rα^−/lox^-CAC mice do not appear to play a protective role in colon tumorigenesis. (**A**) Representative dot plots and graphs of total percentages of CD4^+^ and CD8^+^ T cells in MLN. (**B**) TNF-α, IFN-γ and IL-10 production by 1 × 10^5^ spleen cells stimulated with plate-bound anti-CD3 antibody for 48 h. Cytokines were analyzed in supernatants using a CBA Mouse Inflammation Kit. (**C**) Representative and total RT–PCR data for IL4Rα, Arginase-1, IL13Rα2 and iNOS from colon tissue samples normalized with the GAPDH constituent gene. (**D**) H&E staining to determine architecture and structure of the colon epithelium, as well as Alcian blue staining to determine mucus production by goblet cells. (**E**) Confocal representative merged image of immunofluorescence staining of colon tissue of WT CTRL, WT-CAC, IL4RαKO-CAC and LysMcreIL4Rα^−/lox^-CAC mice using DNA-binding dye (DAPI) in blue and pSTAT6 green. Photographs were taken with a 63× objective. The data came from three different experiments with at least three mice per group. A one-way ANOVA was performed for all the panels and the mean was presented with standard deviation for each experimental group. *p* ≤ 0.05 (*), *p* ≤ 0.01 (**), *p* ≤ 0.0001 (****). Scale bars indicates 10 μm.

Subsequently, different immunological parameters were evaluated in order to better define the possible role of colonic infiltrated M1 macrophages in LysMcreIL4Rα^−/lox^-CAC mice. We did not find differences in the percentage of CD4^+^ T cells in MLN (Figure 5A) compared to IL4Rα-CAC mice. However, the percentage of CD8^+^ T cells decreased in LysMcreIL4Rα^−/lox^-CAC mice to similar levels to those of WT-CAC mice (Figure 5A). Also, LysMcreIL4Rα^−/lox^-CAC mice showed low IFN-γ production (Figure 5B). Therefore, we decided to evaluate whether the changes in the percentage of T cell populations in LysMcreIL4Rα^−/lox^ CAC mice could be related to other cytokines, such as IL-10, an anti-inflammatory cytokine with effects in immunoregulation, and TNF-α, an inflammatory cytokine involved in the activation and proliferation of naïve and effector T cells [29]. We found that LysMcreIL4Rα^−/lox^-CAC mice displayed low levels of these cytokines, while IL4RαKO-CAC mice displayed a higher production of both IFN-γ and TNF-α as well as IL-10 (Figure 5B). Next, we evaluated whether the above results in LysMcreIL4Rα^−/lox^-CAC mice could be related to the expression of IL4Rα in other cell types. We found that both LysMcreIL4Rα^−/lox^-CAC mice and WT-CAC mice overexpressed IL4Rα in colon tissue (Figure 5C). Consistent with this observation, the expression of Arg1 was also higher in LysMcreIL4Rα^−/lox^-CAC mice than in IL4RαKO-CAC mice (Figure 5C). Consequently, we considered whether the elevated expression of Arg1 in LysMcreIL4Rα^−/lox^-CAC mice could also be associated with the expression of the type 2 monomeric receptor of IL-13 (IL13Rα2). Our findings indicate that LysMcreIL4Rα^−/lox^ CAC mice did not express IL13Rα2 (Figure 5C). Therefore, the elevated expression of Arg1 in LysMcreIL4Rα^−/lox^-CAC mice was promoted by IL4Rα and not by IL13Rα2, while no differences were found in iNOS gene expression in the colon (Figure 5C). Moreover, LysMcreIL4Rα^−/lox^ CAC mice displayed greater damage in the structure and architecture of the colon epithelium (Figure 5D), as well as a decrease in mucus production by goblet cells (Figure 5D) which could be associated with the high expression of IL4Rα in LysMcreIL4Rα^−/lox^-CAC mice in colon tissue and with the phosphorylation of STAT6 (pSTAT6) [30]. In contrast, in IL4RαKO-CAC mice, as expected, pSTAT6 was not observed (Figure 5E; Supplementary Figure S5). These results suggest that colon infiltrated M1 macrophages in LysMcreIL4Rα^−/lox^-CAC mice were not enough to exert a protective role during CAC.

## 3. Discussion

Currently, it is widely accepted that an inflammatory microenvironment is one of the main risk factors associated with the development of colitis-associated colon cancer (CAC). Nevertheless, anti-inflammatory responses mediated by IL-10 or the IL-4/STAT6 signaling pathway can favor those immune cells infiltrated into the tumor to acquire protumoral phenotypes such as tumor-associated macrophages (TAMs), which fuel the development and establishment of different types of tumors [9,31,32]. In this study we confirmed that the complete absence of IL4Rα during CAC was directly related to a significant reduction in the number of colonic tumors. Additionally, this finding was apparently associated with the prevalence of an inflammatory response in IL4RαKO-CAC mice, characterized by maintaining a normal percentage of CD8^+^ T cells, an increased IFN-γ and TNF-α production and a higher infiltration of M1 macrophages (F480^+^TLR2^+^STAT1^+^) overexpressing iNOS in the colon. Several studies have confirmed that iNOS expression in macrophages is necessary to eliminate tumor cells [33,34,35], since iNOS is able to improve chemo-sensitization to cisplatin cytotoxicity [15], induce apoptosis [36] and inhibit tumor cell growth [35,36,37]. In the immune system has been reported that iNOS expression can be induced by IFN-γ, an inflammatory cytokine produced by CD8^+^ [38] and CD4^+^T cells, which contributes to the activation of tumoricidal macrophages [25,38,39]. Indeed, although in IL4RαKO-CAC mice the inflammatory response in the colon could be capable of generating damage in surrounding tissue due to the infiltration of M1 macrophages and iNOS expression, we did not find damage in mucus-producing goblet cells, nor did we find damage to the structure and architecture of the colonic epithelium. In fact, IL4RαKO-CAC mice overexpressed Arg1 and Relmα1, which are typical genes that promote tissue-damaged repair caused by early inflammatory processes [27]. Consistent with our results, in previous studies using STAT6 deficient mice (STAT6^−/−^)—STAT6 being the main transcription factor downstream of IL4Rα—no significant reduction of Arg1 and Ym1 expression was observed in DSS-challenged STAT6^−/−^ mice [40]. However, in our work we found that overexpression of Arg1 and Relmα1 in IL4RαKO-CAC mice could be related to other anti-inflammatory pathways, such as those mediated by IL13Rα2 expression in colon, which is the type 2 monomeric receptor of IL-13. In the past, it was thought that IL13Rα2 acted only as a decoy receptor, but it has been shown that IL-13 can signal through IL13Rα2 in a STAT6 independent pathway [41] and is involved in epithelial cell regeneration in the gastrointestinal tract [42]. In this way, our data suggest that during CAC development, the global absence of IL4Rα generates an inflammatory response that favors the recruitment of M1 macrophages (F480^+^TLR2^+^STAT1^+^iNOS^+^) in the colon and that this could be associated with lower rates of tumor development through IFN-γ production by T cells which may be orchestrating tumoricidal responses, while the IL13Rα2 receptor could be promoting tissue repair processes through Arg1 and Relmα1 as a compensatory mechanism in the absence of IL4Rα. There are data indicating a protective role for IFN-γ and TNF-α in colon cancer, for example, in a murine model of CAC, IFN-γ deficient mice (IFN-γ^−/−^) developed both larger number of tumors and higher levels of anti-inflammatory cytokines than wild-type mice [43,44]. In the APC^min/+^ IFNγ^−/−^ murine model, it was shown that the absence of IFN-γ induced higher number of adenomas and consequently approximately 50% of mice developed adenocarcinomas [44]. Similarly, in a DSS-induced experimental colitis model, TNF-α decreases tumor damage by promoting healing through the Wnt/β-catenin signaling pathway [45,46,47]. Likewise, it has been suggested that the systemic production of TNF-α protects against the spontaneous development of colitis and CAC [48]. Nevertheless, there are also controversial reports about the role these proinflammatory cytokines play as tumor promoters; for example, low levels of IFN-γ may enhance the survival of tumor cells, such as prostate cancer cells and lung cancer cells [49]. Furthermore, TNF-α produced during early stages of inflammation has been described in the origin, development, survival and promotion of tumor growth in either CAC or CRC [50,51]. 

**Figure 6:**
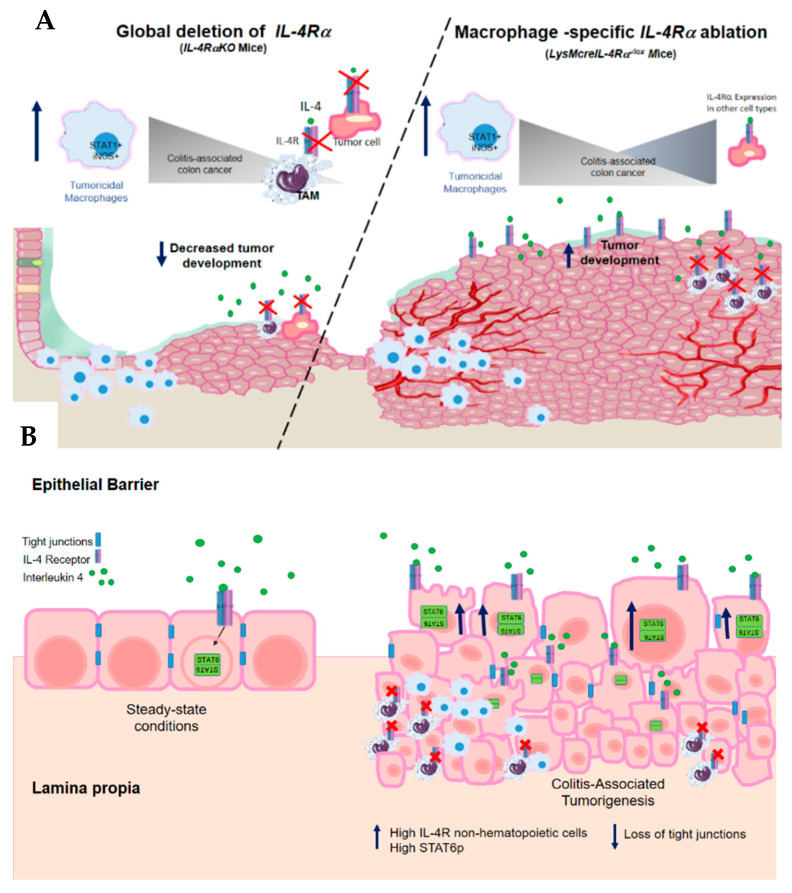
Model showing the possible IL-4Rα-dependent modulation on CAC development. (**A**) In both the global deletion of IL4Rα and the macrophage-specific IL4Rα deletion there is a high recruitment of M1 macrophages in the colon. However, the CAC outcome provides a contrast. Whereas global IL4Rα KO mice do not develop colon tumors, macrophage-specific IL4RαKO mice develop colon tumors similar to WT mice. (**B**) Hypothetical model showing how the expression of IL4Rα and the overactivation of STAT6 can favor the development of CAC through the loss of tight junctions in the colonic epithelium. In both situations, the recruitment of M1 macrophages appears not to be critical for the elimination of CAC.

For several years it has been consistently documented that certain macrophages, such as TAMs, have protumoral roles in different types of cancer [9,10,20]. For this reason, new therapies have focused on both targeting the blockade of IL4Rα in TAMs [52] and on the suppression of M2 polarization to enhance M1 macrophage activity, for example, by blockade of IL4Rα in TAMs along with administration of zoledronic acid, induced apoptosis and delayed breast tumor progression [19,53]. In contrast, in our work we found that although LysMcreIL4Rα^−/lox^ mice do not have macrophages expressing IL4Rα that favor TAM phenotype activation during CAC, these mice developed tumors and sustained greater damage to the structure and architecture of the intestinal epithelium, and also had lower rates of mucus production. In fact, although LysMcreIL4Rα^−/lox^-CAC mice displayed a greater infiltration of M1 macrophages and iNOS expression into the colon, these macrophages seem no longer to have a tumoricidal and protective role that is associated with decreased percentages of CD8^+^ T cells and lower IFN-γ and TNF-α production [29,54,55], suggesting a weak inflammatory immune response. Additionally, colons of LysMcreIL4Rα^−/lox^-CAC mice displayed decreased IL13Rα2 expression while up-regulated IL4Rα expression, which correlates with the observations in WT-CAC mice. We do not know the specific time for the pathological transformation of the IL-4/STAT6 signaling pathway favoring CAC development such that it is detrimental to the immune response. In fact, during steady-state conditions, IL4R downstream transcription factor STAT6 promotes the proliferation and differentiation of secretory intestinal epithelial cells (IECs) in normal colon tissue [56]. Not only that, STAT6 overactivation in IECs has been associated with loss of the tight junctions that leads to permeability dysregulation, bacterial translocation and changes in the microbiome, as well as intestinal inflammation and tumorigenesis [57]. In line with these observations, previous studies support the hypothesis that STAT6 is critical in the early steps of CAC development, and mice with global deletion of STAT6 exhibited significantly reduced numbers of colon tumors [30]. Consistent with this finding we found that colonic tissue of both LysMcreIL4Rα^−/lox^-CAC and WT-CAC mice showed higher phosphorylation of STAT6 (pSTAT6), but, interestingly, IL4RαKO-CAC mice did not display pSTAT6 expression in colon tissue. Therefore, is likely that in LysMcreIL4Rα^−/lox^-CAC mice the immune response mediated by M1 macrophages was not sufficient to stop tumor growth due to IL4Rα expression and pSTAT6 overactivation in colon tissue. Thus, IL4Rα expression in non-hematopoietic cells, such as epithelial cells, may promote colon tumorigenesis in the absence of IL4Rα on macrophages in LysMcreIL4Rα^−/lox^-CAC mice. Supporting this observation, a recent study with the AOM/DSS model has shown that in an IEC restricted loss of IL4Rα (IL4Rα^ΔIEC^mice) tumors were significantly smaller, which correlated with a reduced proliferation detected by BrdU incorporation, although non-immunological parameters were analyzed [40]. However, typically, IL-4Rα is not expressed or is poorly expressed in normal epithelial tissues (as shown here in the colons of healthy WT mice) but it is overexpressed on the surface of many solid tumors, where IL-4 can stimulate angiogenesis through a soluble VCAM-1/alpha4 integrin pathway from endothelial cells [58,59], while in the immune system elevated levels of IL-4 (normally produced by tumor-infiltrating lymphocytes) may contribute to apoptosis resistance of solid tumors [60,61]. Additionally, IL-4 also supports enhanced proliferation and survival of cancer cells in part by inducing glucose uptake [62,63]. In this sense, our results suggest that the significant reduction of colon tumors in IL4RαKO could be associated with an inflammatory response. However, we also found that the expression or non- expression of IL4Rα in macrophages and a TAM phenotype in CAC were not the main factors responsible for the initial changes leading to the promotion of colon tumor development (Figure 6). 

Extensive attention has been paid to the role of TAMs on different types of cancers. Here, we demonstrated that both TAMs or M1 macrophages are dispensable for AOM/DSS induced colon tumorigenesis. Our data suggest that it is likely that IL4Rα expression in other cell types (non-immune cells), as well as the cytokines and cells infiltrated into the tumor microenvironment, replace the pro-tumoral activity mediated by TAMs. Further studies are definitively needed to determine the time of overexpression of IL4Rα on IECs during colon tumorigenesis, which may allow for the design of new immunotherapies targeting IL4Rα at specific times and in specific cells to increase the effectiveness of treatments, instead of targeting whole M1 or M2 macrophage populations.

## 4. Materials and Methods

### 4.1. Mice

Female mice BALB/c, IL4Rα knock-out (IL4RαKO), IL4Rα specific knock-out under the lysozyme promoter M (LysMcreIL-4R^α−/lox^) and non-transgenic for CRE (IL-4Rα^−/lox^) 8 to 10-weeks-old were kept in a pathogen-free environment at the animal facilities of Facultad de Estudios Superiores Iztacala (FES-I), Universidad Nacional Autónoma de México (UNAM). All experimental procedures were in strict accordance with the recommendations in the Guide for the Care and Use of Laboratory Animals of the National Institutes of Health (USA) and were approved by the Committee on Ethics of Animal Experiments, FES-I (UNAM), under number CE/FESI/102016/1096 (18/10/16). IL4RαKO, LysM^cre^IL-4Rα^−/lox^ and IL-4Rα^−/lox^ mice were kindly donated by Dr. Frank Brombacher [64,65].

### 4.2. Generation of LysMcreIL-4Rα^−/lox^ BALB/c Mice

LysMcre mice under lysozyme promoter M were first cross-bred with BALB/c mice for nine generations and then crossed with IL4RαKO BALB/c mice to yield doubly transgenic LysMcreIL4RαKO BALB/c mice. These mice were additionally crossed with IL4Rαlox/lox BALB/c mice to generate LysMcreIL4Rα^−/lox^ BALB/c specific mice. Hemizygocity of IL4Rα (^−/lox^) increases the probability of CRE-mediated removal of the “floxed” allele [64,65].

### 4.3. Induction of Colitis Associated Colon Cancer (CAC) Model

Induction of CAC was performed based on the widely used model of AOM (azoxymethane)/DSS (Dextran sodium sulfate) [66]. Briefly, a single intraperitoneal (i.p.) dose of AOM (Sigma, St. Louis, MO, USA) was administered at 12.5 mg per kg of body weight. Five days later, mice received 2% DSS (*M*_W_: 35,000–50,000, MP Biomedicals, Solon, OH, USA) in their drinking water for 7 days. Subsequently, the mice were rested with regular water for 14 days. The cycle with DSS was repeated two more times. The mice were euthanized 2 weeks after ending the third DSS cycle.

### 4.4. Histological Analysis

Distal colon sections were fixed in 100% ethanol, processed through a treatment with alcohols and xylol. Subsequently, the tissues were embedded in paraffin and sections with a thickness of 5 μm were made. Tissue sections were stained with hematoxylin and eosin (H&E) to visualize cell morphology or with Alcian blue to visualize mucus production by goblet cells. An optical microscope (Axio Vert. A1, Carl Zeiss) was used to visualize the stained tissues.

### 4.5. Immunofluorescence (IF)

Four microns thick colon sections were rehydrated through alcohol gradients and incubated with 10x DIVA Decloaker (Biocare Medical; Berry Drive, Pacheco, CA, USA) at a 1:10 dilution. Slides were washed with PBS1x 3 times for 5 min and membrane permeabilization performed with PBS containing 2% Triton (Reasol; Mexico City, Mexico). Sections were washed with PBS1x (3 × 5 min) and blocked with PBS containing 3% BSA for 1 h at room temperature. Different tissue sections were incubated overnight at 4 °C with purified primary antibodies: rabbit anti-Mouse STAT6P (STAT6P, phosphorylate) (Abcam, Van Allen Way, Carlsbad, CA, USA) at a dilution 1:100, rat anti-Mouse F480 (TONBO, San Diego, CA, USA) at a dilution 1:300, rabbit anti- mouse Arg (Cell Signaling, Danvers, MA, USA) at a dilution 1:50, rabbit anti-Mouse iNOS (Cell Signaling) at dilution 1:400. Next, slides were washed with PBS1x 3 times. Subsequently, tissue sections were incubated for 2 h at room temperature with secondary antibodies with fluorescent label goat anti-rat alexa fluor^®^ 647, (Abcam, Van Allen Way, Carlsbad, CA, USA) at a dilution 1:600 and alexa fluor 546 (Thermo Fisher Scientifics, Rockford, IL, USA) at a dilution 1:900. Tissue sections were washed three times with PBS1x for 1 min and dehydrated through alcohol gradient and mounted with one drop of Fluoroshield mounting medium with DAPI (Abcam, Van Allen Way, Carlsbad, CA, USA) per tissue section. Immunofluorescence was analyzed using an LSM710 DUO (Carl Zeiss GmBH, -Promenade, Jena, Germany).

### 4.6. Isolation of Cells from Colon Tumors for Flow Cytometry

The colon was removed, washed with saline solution and cut first longitudinally and then laterally into pieces of approximately 0.5 cm length. Subsequently, we continued with the steps recommended in the MACs Miltenyi biotec tumor disaggregation kit.

### 4.7. Flow Cytometry

1 × 10^6^ cells from spleen, blood, mesenteric lymph nodules and colon tissue were stained with BV421-F4/80, APC-TLR2 and PECy7-PDL1 antibodies (Biolegend^®^, San Diego, CA, USA) and subsequently incubated for 30 min at 4 °C in the dark. The cells were washed twice with 1 mL of FACS Sheat solution (Becton Dickinson, San Jose, CA, USA) and centrifuged at 1800 rpm for 5 min. The supernatant was decanted and the cells were resuspended in 350 µL of FACS Sheat (Becton Dickinson, San Jose, CA, USA). The cells were analyzed on the Attune NxT flow cytometer (ThermoFisher^®^, Rockford, IL, USA) 10,000 events gated in the cell population of interest per sample were captured. Data analysis was performed with FlowJo software V X (Tree Star).

### 4.8. Transcription Factors in Colon Cells for Flow Cytometry

We prepared a cell suspension of 10 × 10^6^ cells of colon tissue per ml in flow cytometry stain buffer. 100 μL of cells per tube with fluorescent antibodies (F480) was incubated for 30 min at 2–8 °C. Subsequently, we continued with the steps recommended in the BD Pharmingen™ Transcription Factor Buffer Set (562574). 

### 4.9. Cell Culture and Cytokine Quantification

Spleen cells and MLN cells were adjusted at 1 × 10^5^ cells/mL, stimulated with anti-CD3 antibody (5 μg/mL per well) coated in 96-well plates and incubated in complete RPMI medium in a humidified atmosphere containing 5% CO2 at 37 °C for 48 h. Supernatants were collected and stored at −20 °C until analysis was required. The supernatant quantification of cytokines was determined using CBA Mouse Inflammation Kit (BD) according to the instructions described by the supplier.

### 4.10. RNA Isolation and RT–PCR

Sections of the colon distal region (0.5 cm) were homogenized in 500 μL TRIzol. RNA was extracted by chloroform technique. cDNA was synthesized from the extracted RNA using the SuperScript ™ First-Strand Synthesis System (Invitrogen, Carlsbad, CA, USA). RT–PCR was performed using the KAPA Taq enzyme (Kapa Biosystems, Woburn, MA, USA). The primers used to amplify the genes are listed in Table 1.

### 4.11. Statistical Analysis

Statistical differences between groups were determined by One-Way ANOVA with Tukey’s Multiple Comparison test. All statistical analyses were performed using PRISM 5 software (GraphPad, San Diego, CA, USA).

## Figures and Tables

**Table 1:** Primers used in this work.

Genes	Forward Primer	Reverse Primer
IL4Rα Wild Type	TGACCTACAAGGAACCCAGGC	CTCGGCGCACTGACCCATCT
IL4Rα Deleted	GGCTGCTGACCTGGAATAACC	CCTTTGAGAACTGCGGGCT
IL4Rα lox	CCCTTCCTGGCCCTGAATTT	GTTTCCTCCTACCGCTGATT
LysMcre	CTTGGGCTGCCAGAATTTCTC	CCCAGAAATGCCAGATTACG
IL13Rα2	ATA CGT ACG CAT TTG TCA GAG CA	CCA AGC CCT CAT ACC AGA AAA AC
Arginase 1	CAGAAGAATGGAAGAGTCAG	CAGATATGCAGGGAGTCACC
Relmα	GGTCCCAGTGCATATGGATGAGACCATAGA	CACCTCTTCACTCGAGGGACAGTTGGCAGC
iNOS	CTGGAGGAGCTCCTGCCTCATG	GCAGCATCCCCTCTGATGGTG
GAPDH	CTCATGACCACAGTCCATGC	CACATTGGGGGTAGGAACAC

## Data Availability

Not applicable.

## Supplementary Materials

The following are available online at https://www.mdpi.com/article/10.3390/ijms222011204/s1.
