# Supplementary material for: Recruitment of M1 Macrophages May Not Be Critical for Protection against Colitis-Associated Tumorigenesis
Source: Int J Mol Sci. 2021 Oct 18;22(20):11204. doi: 10.3390/ijms222011204 (PMC8536994; doi:10.3390/ijms222011204)
Supplement: Supplementary file 1 [file ijms-22-11204-s001.zip › ijms-1401663-supplementary.pdf]

International Journal of *Molecular Sciences*

MDPI

Supplementary Materials:

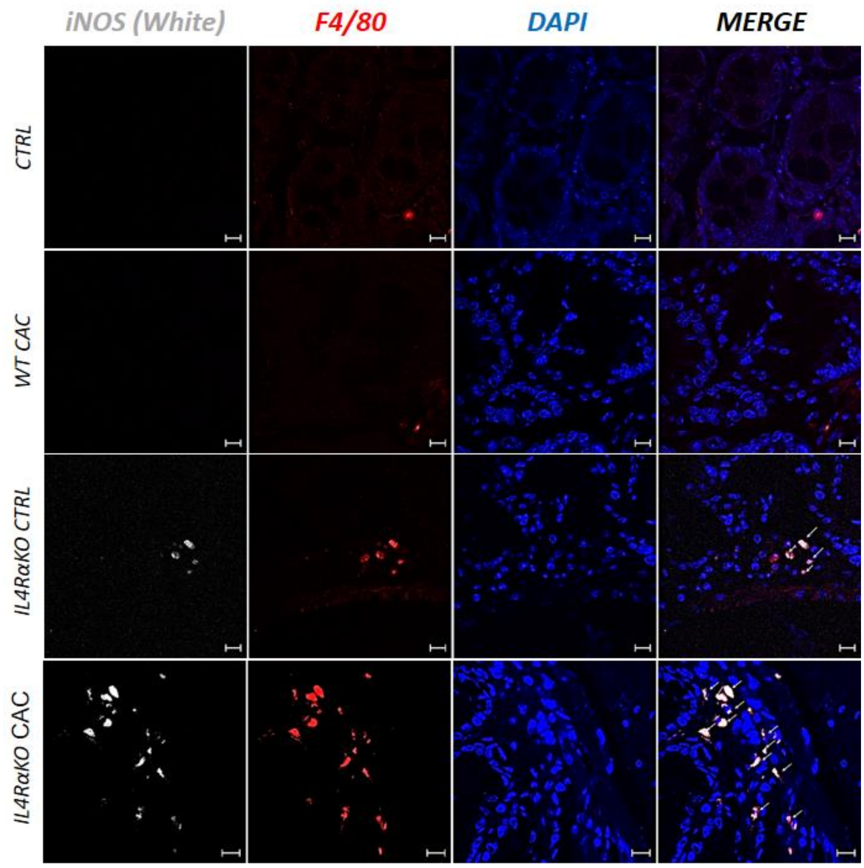

Supplementary Figure S1. Confocal representative M1 macrophages WT CTRL, WT-CAC, IL4RαKO-CAC mice. Confocal representative merged image of immunofluorescence staining using DNA-binding dye (DAPI) in blue, F480 in red and iNOS in white. Photographs were taken with a 63X objective.

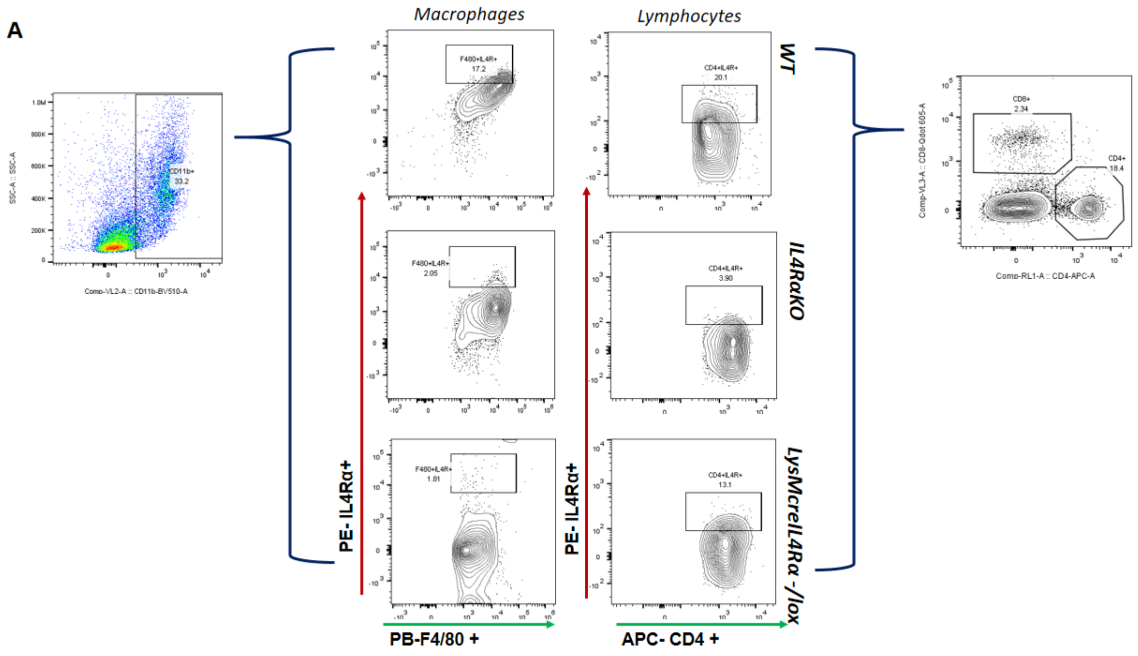

Supplementary Figure S2. Genotyping LysMcreIL4Rα$^{-/lox}$ mice through flow cytometry. Representative dot plots of macrophages (F480^{+}IL4Rα^{+}) and lymphocytes (CD4^{+}IL4Rα^{+}) in colon tissue of groups WT mice, IL4RαKO mice and LysMcreIL4Rα$^{-/lox}$ mice.

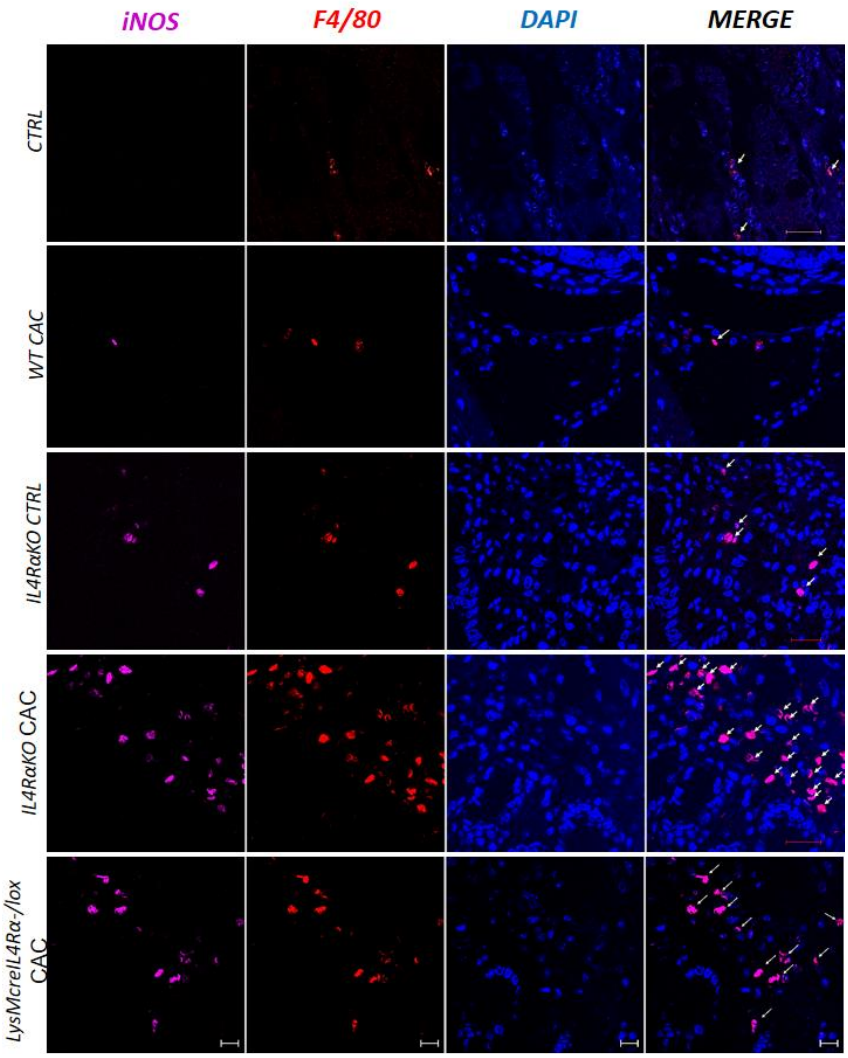

Supplementary Figure S3. Confocal representative M1 macrophages WT CTRL, WT-CAC, IL4RαKO-CAC and LysMcreIL4Rα$^{-/lox}$-CAC mice. Confocal representative merged image of immunofluorescence staining using DNA-binding dye (DAPI) in blue, F480 in red and iNOS in purple. Photographs were taken with a 63X objective.

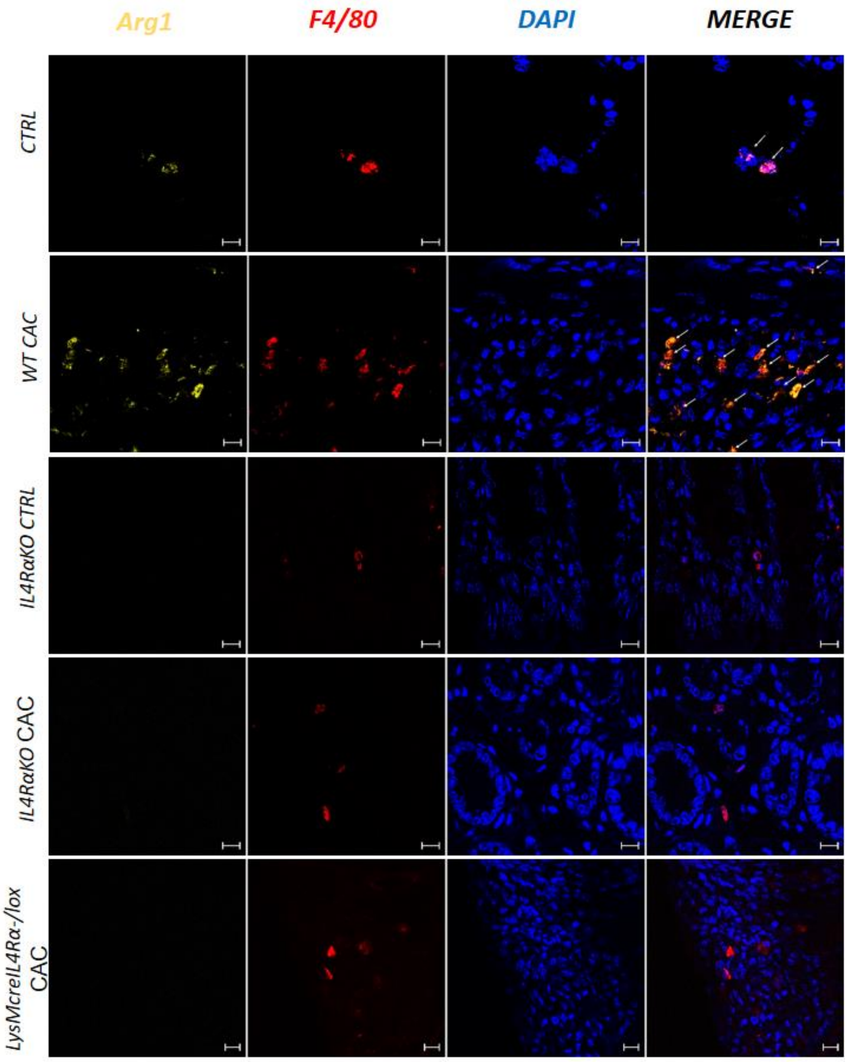

Supplementary Figure S4. Confocal representative M2 macrophages WT CTRL, WT-CAC, IL4RαKO-CAC and LysMcreIL4Rα$^{-/lox}$-CAC mice. Confocal representative merged image of immunofluorescence staining using DNA-binding dye (DAPI) in blue, F480 in red and Arg1 in yellow. Photographs were taken with a 63X objective.

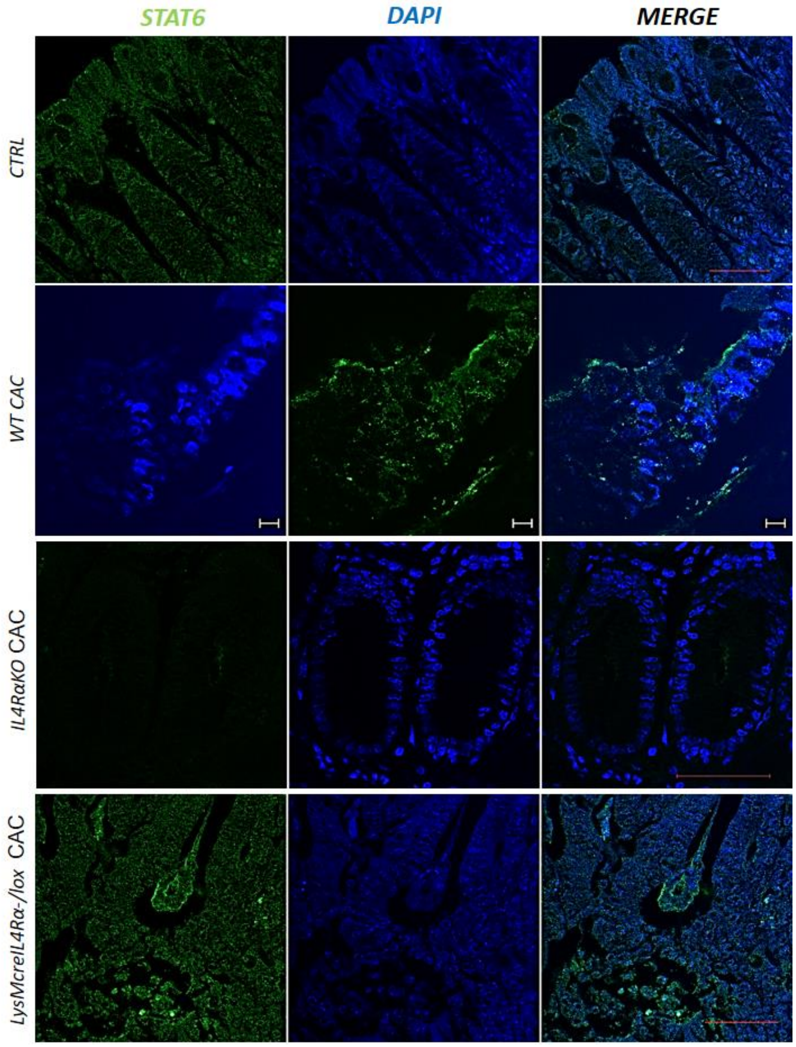

Supplementary Figure S5. Confocal representative pSTAT6 of colon tissue of WT CTRL, WT-CAC, IL4RαKO-CAC and LysMcreIL4Rα$^{-/lox}$-CAC mice. Confocal representative merged image of immunofluorescence staining using DNA-binding dye (DAPI) in blue and pSTAT6 in green. Photographs were taken with a 63X objective.
